# Uncoupling growth from phosphorus uptake in *Lemna*: Implications for use of duckweed in wastewater remediation and P recovery in temperate climates

**DOI:** 10.1002/fes3.244

**Published:** 2020-08-30

**Authors:** Jaimie B. Paterson, Miller Alonso Camargo‐Valero, Alison Baker

**Affiliations:** ^1^ Centre for Plant Sciences School of Molecular and Cellular Biology Faculty of Biological Sciences University of Leeds Leeds UK; ^2^ BioResource Systems Research Group School of Civil Engineering Faculty of Engineering University of Leeds Leeds UK; ^3^ Departamento de Ingeniería Química Universidad Nacional de Colombia Manizales Colombia; ^4^ Present address: The Environment Agency South Preston UK

**Keywords:** duckweed, *Lemna*, nitrogen species, phosphate uptake, photoperiod, temperature

## Abstract

Phosphorus (P) is an essential nutrient for crop growth and the second most limiting after N. Current supplies rely on P‐rich rocks that are unevenly distributed globally and exploited unsustainably, leading to concerns about future availability and therefore food security. Duckweeds (Lemnaceae) are aquatic macrophytes used in wastewater remediation with the potential for nutrient recycling as feed or fertilizer. The use of duckweeds in this way is confined to tropical regions as it has previously been assumed that growth in the colder seasons of the temperate regions would be insufficient. In this study, the combined effects of cool temperatures and short photoperiods on growth and P uptake and accumulation in *Lemna* were investigated under controlled laboratory conditions. Growth and P accumulation in *Lemna* can be uncoupled, with significant P removal from the medium and accumulation within the plants occurring even at 8°C and 6‐hr photoperiods. Direct measurement of radiolabeled phosphate uptake confirmed that while transport is strongly temperature dependent, uptake can still be measured at 5°C. Prior phosphate starvation of the duckweed and use of nitrate as the nitrogen (N) source also greatly increased the rate of P removal and in‐cell accumulation. These results form the basis for further examination of the feasibility of duckweed‐based systems for wastewater treatment and P recapture in temperate climates, particularly in small, rural treatment works.

## Graphical abstract

Growth and P accumulation in duckweed can be uncoupled under short photoperiods and low temperature. Nitrate and neutral pH are optimal for growth and P removal and depletion of intracellular P strongly stimulates P uptake. As conditions for P removal and biomass production differ, we believe this information can be incorporated into prototype designs for duckweed‐based treatments at small sewage treatment works in temperate climates.

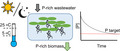

## 1 INTRODUCTION

Phosphorus (P) is an essential nutrient for all living organisms which enters the food chain through its acquisition from environmental sources by primary producers. In approximately 50% of agricultural soils, P is limiting for plant growth and supplemental fertilization is required for optimal crop productivity (Lynch, 2011). However, P‐rich rock required for inorganic phosphate fertilizer production is a limited, nonrenewable resource (Elser & Bennett, 2011; Steiner, Geissler, Geissler, Watson, & Mew, 2015). Paradoxically, a high proportion of P applied as fertilizer is not available to plants due to its fixation in the soil by chemical and biological processes (Bieleski, 1973; Devau, Cadre, Cadre, Hinsinger, Jaillard, & Gérard, 2009) leading to a low efficiency of uptake (Syers, Johnston, Johnston, & Curtin, 2008). Over time, agricultural soil can accumulate P as a result of repeated application, leading to leaching and eutrophication of water bodies (Withers et al., 2017). Additionally, human and animal consumption of food results in elimination of P as feces and urine (Comber, Gardner, Gardner, Georges, Blackwood, & Gilmour, 2013), while other industrial processes also contribute to the production of nutrient‐rich waste waters (Jarvie, Neal, Neal, & Withers, 2006).

In the UK, the production of nearly 11 billion liters of sewage per day contribute to the main source of P loss affecting aquatic ecosystems, despite the fact that water companies comply with an already low P discharge consent (<1 mg P/L). Furthermore, it is expected that more stringent P discharge consents will be imposed to sewage treatment works (STWs; < 0.25 mg P/L), in future, necessitating more costly effluent polishing processes. Raw sewage contains the equivalent of 71% of annually imported P‐fertilizer (Cooper & Carliell‐Marquet, 2013), but the current nutrient control processes at STWs remain a linear approach to nutrient management with limited opportunities for recovery and potential reuse of P as fertilizer. Generally speaking, nearly 50% of all P entering STWs in the UK is recycled to agriculture as sewage sludge, but only 0.8% of the total UK agricultural land area benefits from that practice (Cooper & Carliell‐Marquet, 2013); as a result, the in‐land accumulation of P has recently increased at an average rate of 0.6 kt P/year (Worrall, Jarvie, Jarvie, Howden, & Burt, 2016). Increasing health and environmental concerns due to the presence of heavy metals and persistent and emerging organic pollutants in sewage sludge may accelerate changes in UK policy, as already existing regulations in European countries impose very stringent controls (e.g., Germany) or a ban on this practice (e.g., the Netherlands, Belgium (Flanders region), and Switzerland) (Smith, 2009). Therefore, additional research on alternatives ways to effectively recover and recycle nutrients from sewage into agriculture is needed.

Eutrophication as a result of excessive nutrient levels can cause extensive population increases of macrophytes including duckweed (Lemnaceae) and harvesting of such organisms is suggested as a means of recycling nutrients (Quilliam et al., 2015; Su et al., 2019). Duckweed compete with algae and other aquatic species in the water column by reproducing as rapidly as possible (taking up nutrients as they do) to colonize the surface and shade out the plankton. Under optimal conditions, they have been reported to double in one and a half to four days (Ziegler, Adelmann, Adelmann, Zimmer, Schmidt, & Appenroth, 2015). A number of studies have been reported using duckweed for treatment of different types of wastewater (domestic, agricultural) at different scales and under a wide variety of conditions (Bergmann, Cheng, Cheng, Classen, & Stomp, 2000; Cheng, Bergmann, Bergmann, Classen, Stomp, & Howard, 2002; Cheng, Landesman, et al., 2002; Culley & Epps, 1973; Culley, Rejmánková, Rejmánková, Květ, & Frye, 1981; Hillman & Culley, 1978; Hu et al., 2019; Ishizawa et al., 2020; Ran, Agami, Agami, & Oron, 2004; Xu & Shen, 2011; Zhao et al., 2015; Zhou et al., 2019). However, it is difficult to draw meaningful comparisons given the wide range of experimental designs, scale of implementation (from small pots under controlled conditions to outdoor ponds with fluctuating environmental conditions), and different wastewater characteristics with drastically different nutrient levels. Often the duckweed subspecies/strain/geographical isolate has not been identified even though adaptation to specific conditions may be critical for growth and nutrient removal (Bergmann et al., 2000; Zhao et al., 2015). Further variation may occur due to presence/absence of beneficial microbes which can promote biomass growth and nutrient removal under some conditions (Ishizawa et al., 2020; Toyama et al., 2017).

Duckweeds are distributed throughout the globe in all but the most extreme habitats (Landolt, 1986). However, most reports of nutrient removal by duckweed are conducted in the laboratory under “optimal” temperatures (typically 20–25°C) and photoperiods (12–16 hr) or outdoors in warm climates (El‐Kheir, Ismail, Ismail, El‐Nour, Tawfik, & Hammad, 2007; Ran et al., 2004) and few studies have considered the interactive effect of temperatures and photoperiods experienced during temperate seasons on nutrient removal (Xu & Shen, 2011; Zhao et al., 2015). In this study, we explore the interactions of cool temperatures, short photoperiods, internal P status and N species on P uptake, and accumulation by *Lemna* using controlled nutrient conditions where individual factors can be manipulated alone and in combination, rather than in wastewater which can be variable in composition and therefore more difficult to isolate the effect of individual factors. Our results give insights into some of the factors and trade‐offs required to inform engineering solutions for nutrient control and recovery in STWs in temperate climate countries.

## 2 MATERIALS AND METHODS

### 2.1 Strains and culture medium

Duckweed was collected from a lagoon in North Lincolnshire, UK. Detritus and associated biota were removed by repeated rinses in tap water. Subsamples were transferred to opaque tubs and maintained on autoclaved Hoagland's growth solution described below. The isolate was identified as *Lemna minor/japonica* by sequencing the *atpF‐atpH* noncoding spacer of the chloroplast genome (Wang et al., 2010). This isolate has been deposited with the Rutgers Duckweed Stock Co‐operative (www.ruduckweed.org clone #1053). Experiments were conducted in a Sanyo MLR‐351 growth chamber, in 150 ml volume pots containing 100 ml of solution. With the exception of the experiments presented in Figures 3 and 4, experiments were carried out under nonsterile conditions. Temperature was constant and controlled to ±1°C, and light was provided by fluorescent tubing at 155 µmol photons m^−2^ s^−1^. Temperatures and photoperiods were varied as described for specific experiments in the relevant figure legends. Experimental pots were slotted into a dark‐box to facilitate overhead illumination only. Experiments were conducted in triplicate adopting a daily destructive sampling regime using 3 g initial fresh mass of duckweed per pot.

The Hoagland's solution used for Lemna stock maintenance consisted of: Ca(NO_3_)_2_·4H_2_O (543 mg/L), MgSO_4_·7H_2_O (247 mg/L), KH_2_PO_4_ (68 mg/L), KNO_3_ (253 mg/L); H_3_BO_3_ (2.86 mg/L), NaMoO_4_·2H_2_O (0.025 mg/L), MnSO_4_.5H_2_O (0.025 mg/L), ZnSO_4_·7H_2_O (0.025 mg/L), CuSO_4_·5H_2_O (0.025 mg/L), FeCl_3_.6H_2_O (0.05 mg/L), and ethylene‐diamine‐tetra acetic‐acid (EDTA) (0.75 mg/L) (Hoagland & Arnon, 1950). Solutions were autoclaved before use. Prior to all experiments excluding those investigating phosphate acclimation and nitrogen species, all experimental duckweed inoculums were maintained for a minimum of four days on 15 mg P/L (refreshed daily) before the start of each experiment.

To counter evapotranspiration, experimental solution was topped up each day back to 100 ml with Hoagland's solution including all nutrients listed above except phosphate, and pH was fixed to 7 at the start of all experiments but not buffered.

### 2.2 Experimental set ups

To obtain axenic cultures for radioisotope and mass balance experiments (Figures 3 and 4), using aseptic technique, duckweed roots were removed with a razor blade close to the frond base and immersed for two minutes in 10% (v/v) bleach/dH_2_O to kill epiphytic microbial assemblages. Single fronds were then transferred to 10 ml autoclaved Hoagland's solution in six‐well Repli‐dishes and covered. Fronds were transferred to fresh sterile solution every two days using aseptic technique until enough fronds had reproduced for experiments.

#### 2.2.1 ^32^P transport experiments

Axenic cultures of *Lemna* were maintained on modified Hoagland's solution containing 0.1 mM P for one week prior to experiments. Triplicate 10–20 mg (FM) samples were inoculated into 5 ml of experimental perfusion solution containing 0.1 mM P as KH_2_PO_4_, 0.1 mM MgSO_4_·7H_2_O; 1.8 mM Ca(NO_3_)_2_·4H_2_O and 2.5 mM KNO_3_, fixed to pH 5.7 with 1 mM HEPES buffer (Ullrich‐Eberius, Novacky, Novacky, & van Bel, 1984); and acclimated for 30 min to each experimental temperature of 5, 15, or 25°C. Once acclimated, cultures were spiked with ^32^P (as H_3_^32^PO_4_, Perkin Elmer, UK, specific activity 285.6 Ci/mg) to a working concentration of 1 µCi/mL and incubated for 60 min at each respective temperature. Uptake was halted at 0, 10, 30, and 60 min by rinsing the fronds 3× in dH_2_O, once in 1 mM phosphate buffer (Hase, Nishikoori, Nishikoori, & Okuyama, 2004) and immersing into Opti‐Fluor scintillation fluid (Perkin Elmer, UK), before the plants were counted for radioactivity in a scintillation counter. Uptake data were corrected for decay (0.953), and counts per minute were converted to µmol P mg^−1^ (FM) h^−1^.

#### 2.2.2 Manipulation of internal P

Prior to experiments shown in Figure 5, duckweed stocks were maintained for four days on 0, 1, 2, 5, or 15 mg P/L refreshed daily. To alter the concentration of P, volumes of KH_2_PO_4_ were reduced accordingly and the deficit of K was made up with the addition of KCl to maintain key nutrient balances. Three (3) g (FM) subcultures of *Lemna* (taken from the stocks described above) were inoculated in triplicate into 100 ml of modified Hoagland's solution containing 0, 1, 2, 5, or 15 mg P/L and observed for four days. Following this, the same cultures were re‐inoculated into fresh solutions all containing 15 mg P/L and observed for a further four days. Growth conditions were 12‐hr photoperiods at 15°C and 6‐hr photoperiods at 8°C. P *in planta* was measured daily for the whole eight‐day period and P in solution and fresh mass (FM) were measured daily from days four to eight in destructive samples.

#### 2.2.3 Effect of Nitrogen species and pH

For the experiment shown in Figure 6, one set of media had 15 mg N/L as either ammonium or nitrate and 5 mg P/L (an N:P ratio of 3) and were set to pH 4 initially but unbuffered. The other set had 50 mg N/L as either ammonium or nitrate and 15 mg P/L (N:P ratio of 3.33) and the starting pH set to 7 but unbuffered.

### 2.3 Analytical methods

#### 2.3.1 P analysis

Pots were agitated with a sterile glass rod to mix the growth solution before a 1 ml sample was removed and syringe filtered (0.45 µm Ø pore size, Fisher Scientific, UK). This was diluted when required and the assay conducted follows the methods of Pierzynski (2000).Total and inorganic phosphate in dried plant tissue were determined following the methods of Ames and Dubin (1960) and Chen, Toribara, Toribara, and Warner (1956) respectively.

#### 2.3.2 Fresh and dry mass

Fresh mass (FM) was ascertained by pouring the pot contents through a sieve and gently blotting the duckweed on paper towels before weighing on an analytical balance.

Dry mass (DM) was obtained by drying the fresh duckweed samples in an oven at 70°C for two days in sterile, phosphate‐free glass jars before weighing on an analytical balance.

#### 2.3.3 Data analysis

Phosphate removal coefficients (*K*) were calculated by transforming the mean daily phosphate in solution (*n* = 3) to natural logarithm and plotting against time. Pearson's correlations, *t* tests, and one‐way ANOVA tests for significance were carried out using SPSS (v. 22, IBM, US). Phosphate uptake kinetics (*K*_m_ and *V*_max_) were assumed to follow first order Michaelis–Menten terms (Equation 1) and were calculated by transformation using the Lineweaver–Burk equation (Equation 2).

(1) $$V=V_{\max}*\left[S\right]/K_{m}+\left[S\right]$$

(2) $$1/V=K_{m}/V_{\max}*1/\left[S\right]+1/V_{\max}$$

where *V* = initial phosphate uptake rate; *V*_max_ = maximal P uptake rate; *K*_m_ = P concentration at which 50% of *V*_max_ is achieved; [S] =substrate (P) concentration.

## 3 RESULTS

### 3.1 The effect of varying photoperiod at 15°C on P removal, accumulation, and growth of *Lemna*

**FIGURE 1:**
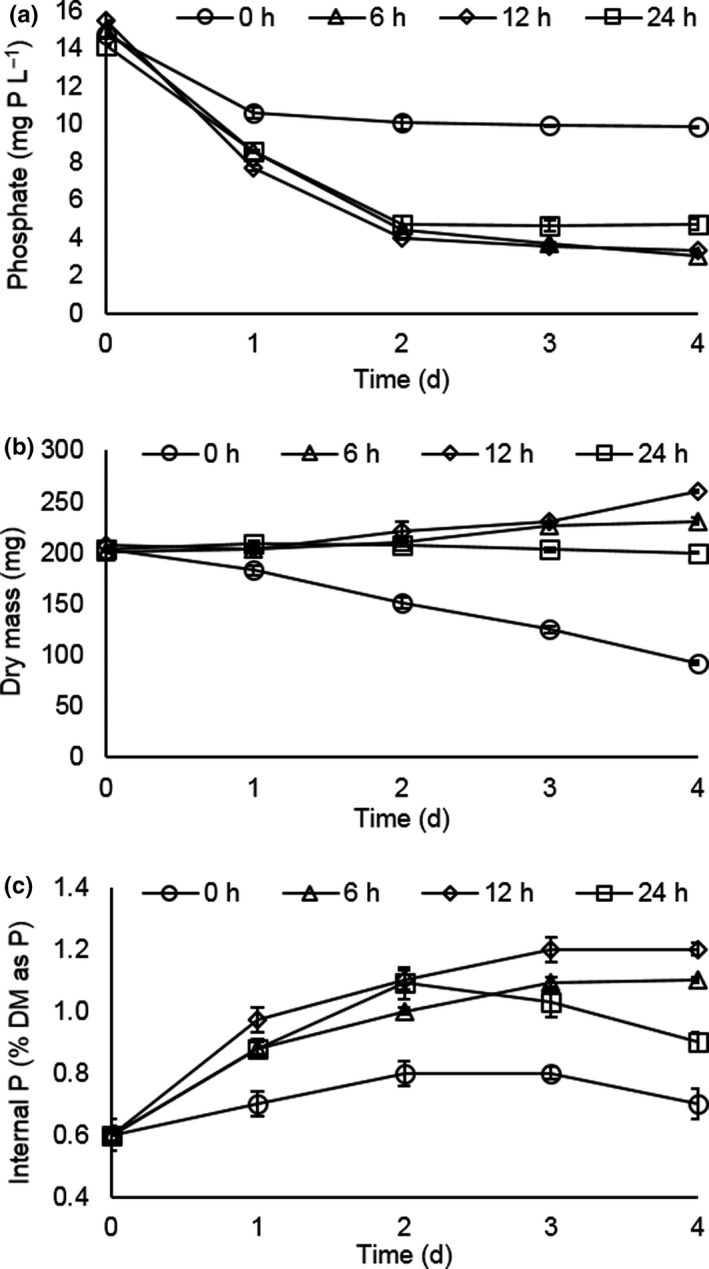
The effect of photoperiod on P removal, accumulation, and growth of *Lemna* under temperate climatic conditions. (a) P removal from solution as a function of photoperiod. (b) Total dry mass as a function of photoperiod. (c) Internal inorganic P (Pi) as a % of total dry mass under varying photoperiod. Error bars are standard error of the means (*n* = 3)

To evaluate the potential of duckweed for nutrient recovery under temperate climate conditions, the effect of varying photoperiod from 0 to 24 hr at a constant temperature of 15°C (which represents UK summer time day plus night average; MET Office, 2013) was investigated. Figure 1a shows that P was removed from solution under all conditions. Plants maintained in darkness removed P over the first 24 hr but then ceased and the P level in solution remained stable at around 11 mg/L. The plants grown under photoperiods of 6, 12, or 24h removed P at a similar rate over the first two days of the experiment. Thereafter the plants maintained in 24‐hr light ceased P removal whereas plants grown in the 6‐hr or 12‐hr photoperiod continued to remove P more slowly than over the preceding two days. Dry mass was used as a proxy indicator of plant growth. Figure 1b reveals that the plants maintained in darkness (0 hr) lost dry mass over the course of the experiment. Plants grown in 24‐hr light maintained their dry mass but did not grow. The plants maintained in 6‐hr and 12‐hr photoperiods showed small increases in dry mass. Regardless of the photoperiod, all plants increased internal Pi content over the first two days of the experiment. Thereafter the plants maintained in 24‐hr light or total darkness showed a decline in Pi content whereas plants in 6‐hr or 12‐hr photoperiods continued to accumulate Pi and the level of accumulation was slightly higher under 12‐hr photoperiod than 6‐hr photoperiod.

### 3.2 The effect of varying temperature on P removal, accumulation, and growth of *Lemna*

#### 3.2.1 The effect of varying temperature under a 6‐hr photoperiod

**FIGURE 2:**
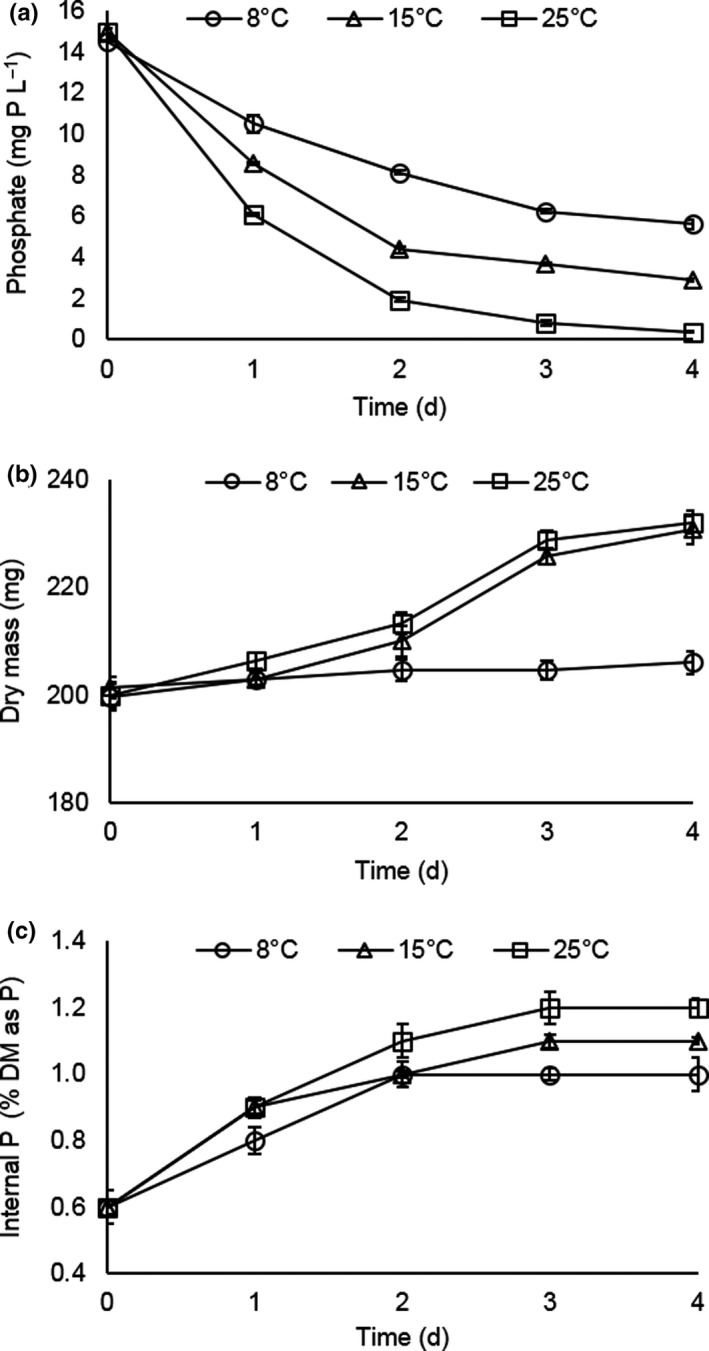
The effect of varying temperature under a 6‐hr photoperiod on P removal, accumulation and growth of *Lemna*. (a) Temperature dependence of P removal from solution. (b) Total dry mass accumulation as a function of temperature. (c) temperature dependence of internal inorganic P (Pi) Error bars are standard error of the means (*n* = 3)

To investigate whether duckweed mediated P remediation could occur under conditions similar to those experienced in northern temperate spring and autumn, the photoperiod was fixed to 6‐hr and the temperature fixed to 8, 15, or 25°C. The latter is close to the reported optimum for photosynthesis in duckweed (Wedge & Burris, 1982). The duckweed removed P from solution at all temperatures (Figure 2a), but unsurprisingly P removal was strongly temperature dependent. At 25°C, the duckweed removed essentially all the P from solution in four days, but even at 8°C, more than half the P was removed. At 15 and 25°C, the duckweed showed similar increases in dry mass over the course of the experiment but the plants maintained at 8°C did not grow (Figure 2b). The internal Pi concentration increased for all temperatures (Figure 2c). It doubled from 0.6% to 1.2% of dry weight in the plants maintained at 25°C but still increased to nearly 1% in the plants maintained at 8°C. Thus, duckweed can continue to remove P from solution and accumulate it intracellularly under short days and cold temperatures, even when conditions are unable to support biomass increase.

#### 3.2.2 Mass balance at different temperatures and the effect of phosphate loading

**FIGURE 3:**
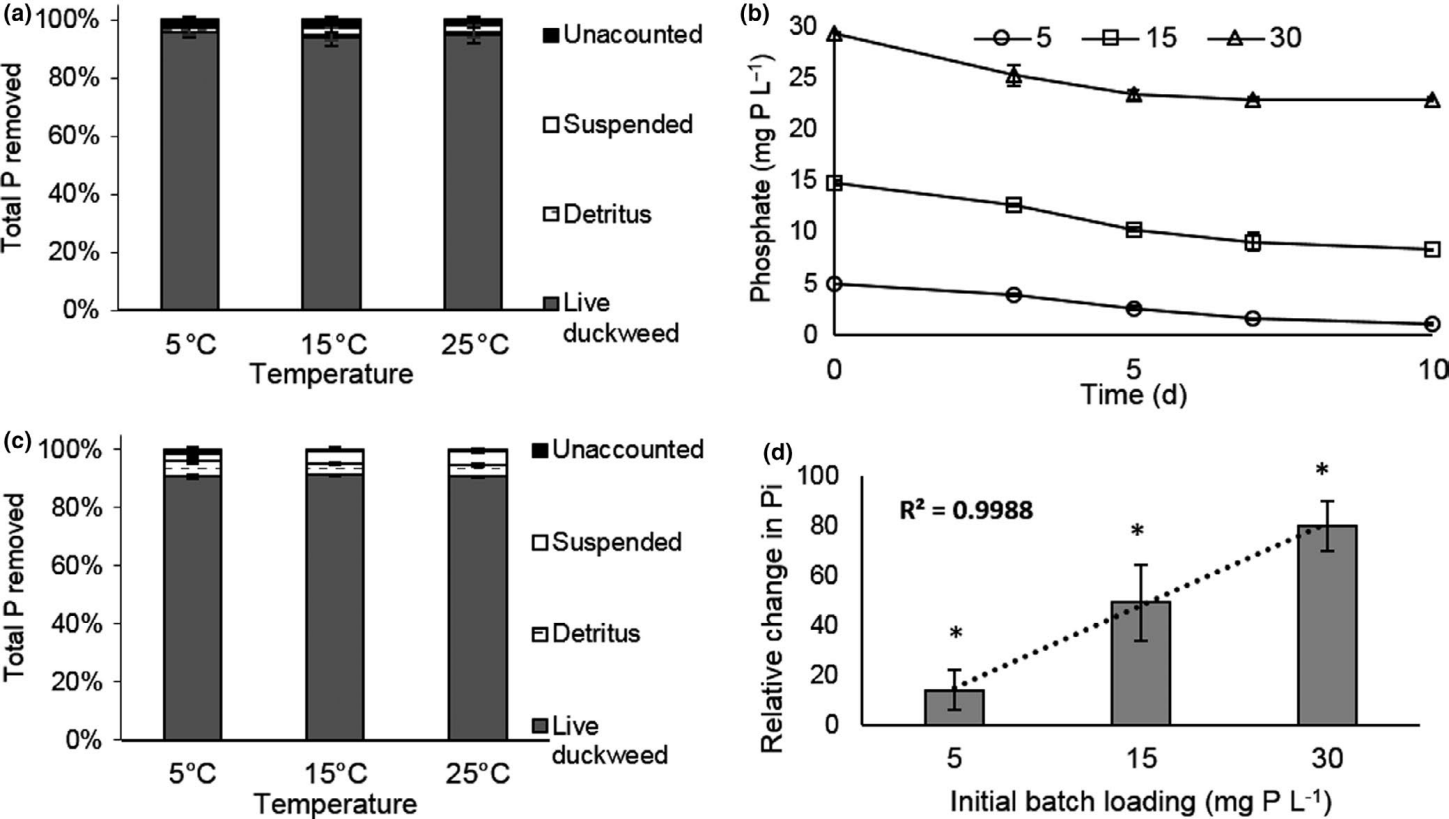
P uptake by *Lemna* and mass balance at varying temperatures and P concentrations. (a) Mass balance for axenic *Lemna* cultures (12‐hr photoperiod, 15 mg P/L) at 5, 15, or 25°C. Total P was measured at day 0 and day 4 in suspended matter, detritus, and in living duckweed. (b) Rate of removal of P from solution as a function of initial P concentration. (c) As (a) but with nonaxenic cultures. (d) Internal inorganic P (Pi), measured at day 0 and day 10 and the % change presented. R^2^ value is the correlation between increase in Pi (relative %) and initial P load (mg P/L). *Indicates a significant difference in relative change between all batch loadings. Error bars are standard error of the means (*n* = 3) for all panels

To be certain that the P being removed from solution was in fact being removed by duckweed and to ensure the efficient recovery of P in the experiments, a mass balance was conducted for axenic (Figure 3a) and nonaxenic (Figure 3c) batch systems over a period of 4 days under a 12‐hr photoperiod at varying temperatures (5, 15, and 25°C), with an initial P load of 15 mg Pl^‐1^. In both systems, close to 100% of P was accounted for. In the axenic system, >94% of total P removed at all three temperatures, and in the nonaxenic cultures, >90% of P removed was recovered in duckweed. Therefore, biological P uptake by duckweed was the dominant mechanism of removal, even in nonaxenic cultures.

To investigate the effect of phosphate loading in this system, we selected a 12‐hr photoperiod and 15°C and varied the initial P concentration (5, 15, and 30 mg P L^‐1^; Figure 3b). As expected, P removal was proportional to initial concentration over the first three days of the experiment before reaching a plateau. Internal Pi was measured for each batch of *Lemna,* and the % change from the starting value calculated as a function of P loading (Figure 3d). This shows an excellent correlation of *R^2^* = 0.99.

#### 3.2.3 Temperature and concentration dependence of phosphate transport

**FIGURE 4:**
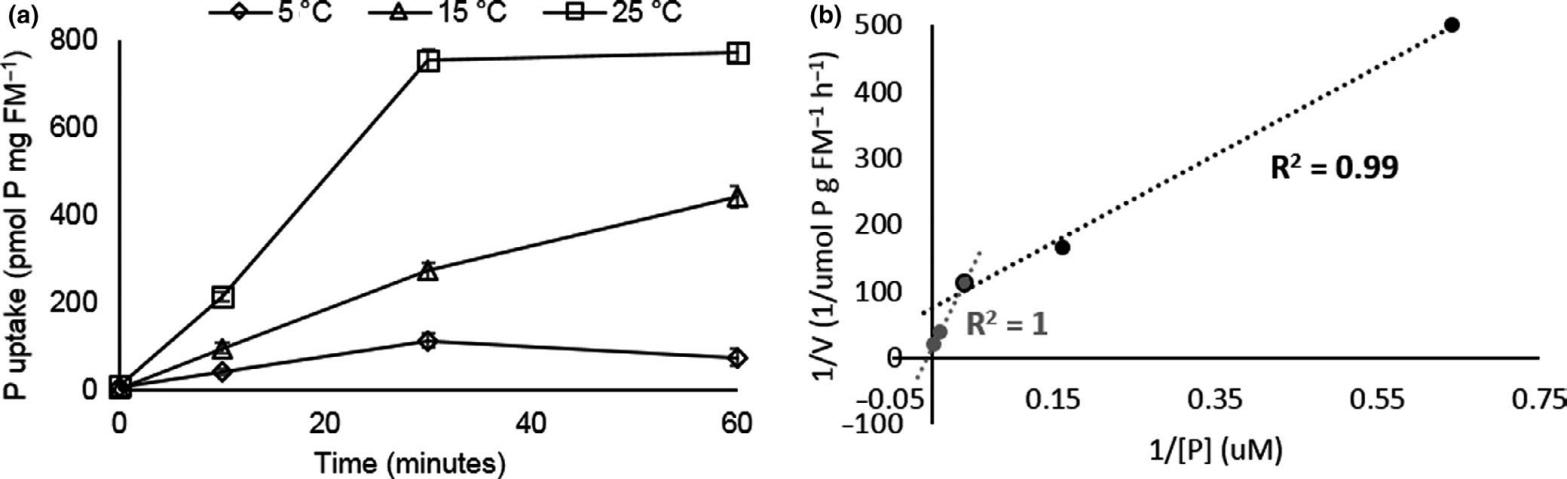
Temperature‐dependent phosphate transport in *Lemna* and biphasic uptake. (a) Temperature dependence of radiolabeled 32P transport by axenic *Lemna* cultures (*n* = 3) (b) Concentration dependence of P uptake. Lineweaver–Burk plot of ^32^P uptake showing low‐ and high‐affinity transport processes. 1.56–25 µM P is in gray; 25–400 µM P is in black

P removal from solution and intracellular storage are the consequence of several steps of transport and metabolism. To understand which steps might be most limiting at low temperature, uptake of ^32^P labeled phosphate from solution as a function of temperature was examined (Figure 4a). Transport was strongly temperature dependent, but even at 5°C, uptake of ^32^P could be measured. The rate of transport was measured as a function of phosphate concentration (0.05 to 12.40 mg P L^‐1^ at 22°C). This showed clear evidence for a biphasic mode of uptake, with a slower high‐affinity rate (*V*_max_ 0.03 µmol PO_4_^3‐^/g FW h^‐1^, *K*_m_ 21 µM) and a faster low‐affinity mechanism (*V*_max_ 21µmol/g FW h^‐1^ *K*_m_ 510 µM) Figure 4b. This is consistent with the more rapid removal of P at higher initial loadings (Figure 3c).

### 3.3 Effect of internal P concentration on P removal by *Lemna*

**FIGURE 5:**
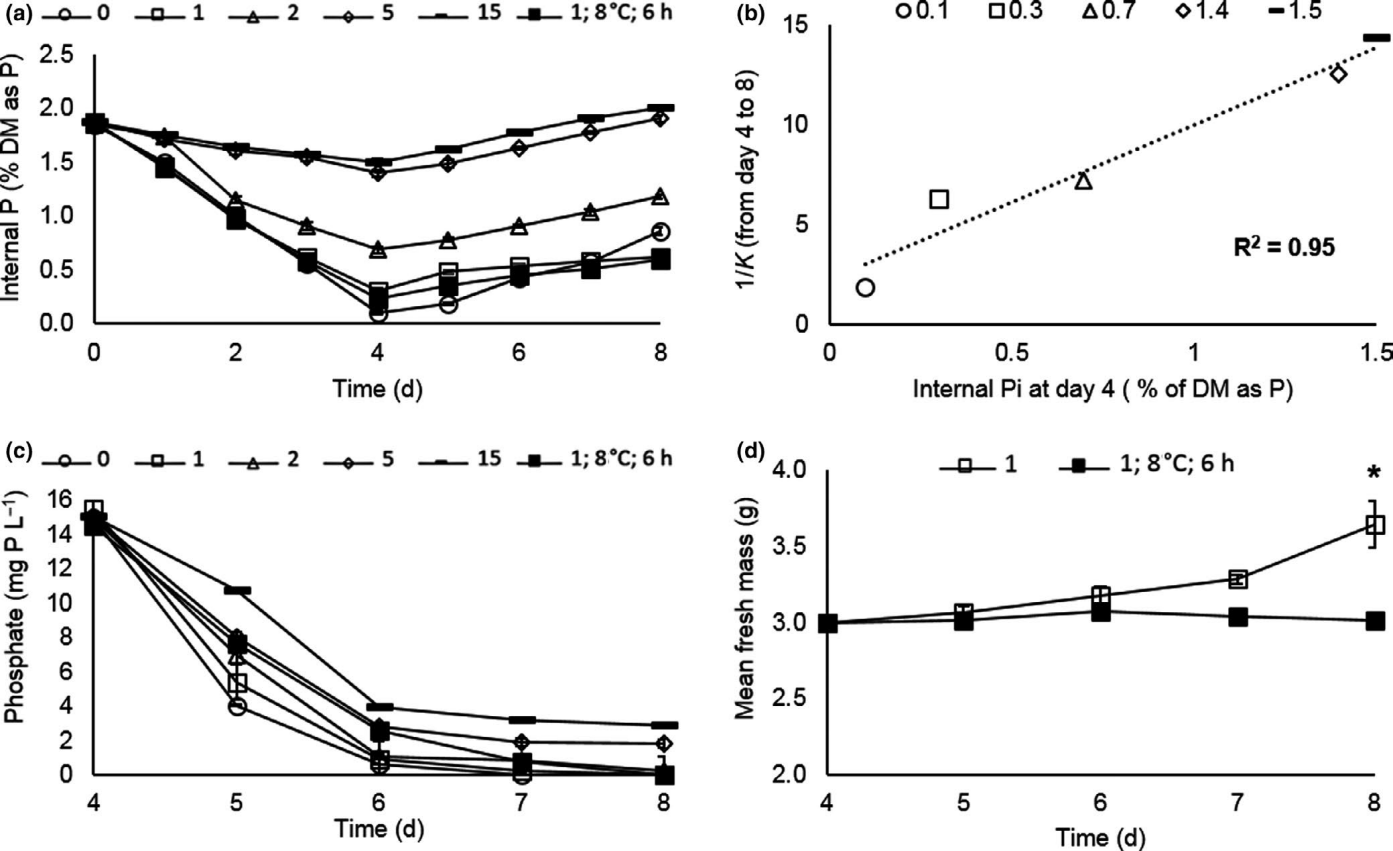
Effect of internal P concentration on P removal by *Lemna*. (a) Internal inorganic P (Pi) as % of dry mass in samples pre‐equilibrated at 0, 1, 2, 5, or 15 mg P/L (0, 1, 2, 5, and 15, respectively) for four days (d0––d4), then transferred to fresh solutions all containing 15 mg P/L (d4–d8) at 15°C, and 12‐hr photoperiod. For comparison, one sample pre‐equilibrated to 1 mg/L P for 4 days was maintained under low temperature and short photoperiod (8°C 6 hr)after transfer to 15 mg/L P. (b) Pearson's correlation of the reciprocals of removal coefficients (*K*) over four days (days 4–8 in Figure 5c) and the respective starting internal P content in cultures measured on day 4. (c) As for A, but P in solution was measured daily from the time of transfer into 15mg L^‐1^ P (day 4). (d) Total fresh mass was measured daily in triplicate destructive samples representative of both growing conditions. “*”indicates a significant difference (*p* = <0.01). Error bars are standard error of the mean (*n* = 3) for all panels

To investigate the effect of pre‐acclimation of the duckweed to different levels of P, cultures were maintained on 0, 1, 2, 5, or 15 mg P L^‐1^ for four days prior to the start of the experiment (Section 2.3). The internal Pi content was monitored (Figure 5a) and showed that the Pi stores were gradually depleted during this pre‐acclimation phase and that the plants maintained on the lowest P solutions (0 and 1 mg P L^‐1^) showed the greatest depletion of internal Pi. Plants maintained on 5 or 15 mg P L^‐1^ showed a much more modest decline. On transferring to 15 mg P L^‐1^, internal Pi pools were replenished (Figure 5a). When the rate of removal of P from the medium was measured, plants pre‐acclimated to the lowest P removed P most rapidly. After four days, all the P had been removed by plants that had been pre‐acclimated to 2 mg P L^‐1^ or less (Figure 5c). The rate of P removal was very strongly correlated with the internal P content of the duckweed at the time of transfer (*R^2^* = 0.95; Figure 5b). Significantly, pre‐acclimation to 1 mg P L^‐1^ enabled plants to remove P from solution rapidly and efficiently even when grown at 8°C under 6‐hr photoperiod. These plants removed all the P in solution after four days and had a similar internal Pi content to plants similarly pre‐acclimated but subsequently grown at 15°C with 12‐hr photoperiods (Figure 5c). However, the plants grown under short day cold conditions did not show increased biomass (Figure 5d) as seen previously (Figure 2b).

### 3.4 Effect of pH and N species on P removal and accumulation by *Lemna*

**FIGURE 6:**
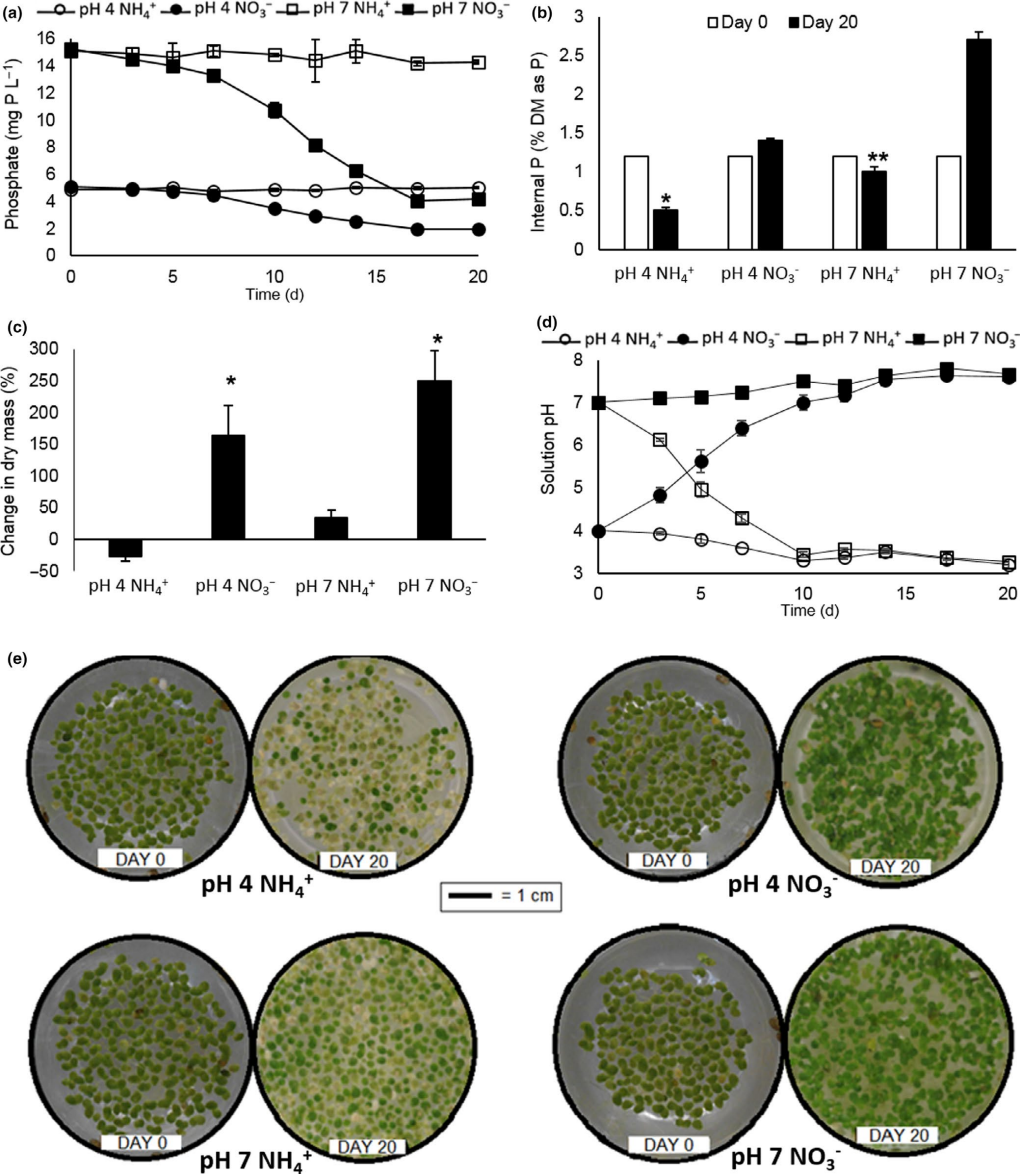
Effect of N species on P removal and accumulation by *Lemna*. (a) Rate of removal of P solution from solutions of four different profiles; 5 mg P/L, 15 mg N (as ${\text{NH}}_{4}^{+}$ or ${\text{NO}}_{3}^{‐}$)L^‐1^, pH 4 (unbuffered); or 15 mg P/L, 50 mg N/L, pH 7. (b) Changes in internal P (as % of dry mass) after 20 days relative to starting values. (c) Changes in the amount of dry biomass produced after 20 days (% relative to starting dry mass). (d) Changes in growth solution pH brought about by the duckweed cultures. Errors are standard error of the means (*n* = 3). “*”indicates a significant difference between the ${\text{NH}}_{4}^{+}$ and ${\text{NO}}_{3}^{‐}$ cultures at pH 4 (one‐way ANOVA, *p* < .01); and “**”indicates a significant difference between the ${\text{NH}}_{4}^{+}$ and ${\text{NO}}_{3}^{‐}$ cultures at pH 7 (one‐way ANOVA, *p* < .01). (e) Phenotypic variations displayed by the cultures after 20 days of incubation in respective solutions

Waste waters vary in the proportion of N species present as ammonium (${\text{NH}}_{4}^{+}$) versus nitrate (${\text{NO}}_{3}^{‐}$). Additionally, there is a lack of consensus in the literature as to which is the preferred N species for *Lemna* sp. (Caicedo, van der Steen, van der Steen, Arce, & Gijzen, 2000; Fang, Babourina, Babourina, Rengel, Yang, & Pu, 2007; Mohedano, Costa, Costa, Tavares, & Belli Filho, 2012; Porath & Pollock, 1981). Therefore, the effect of different N species at different starting pH values on P removal was investigated. The pH is important as it affects the equilibrium between the different forms of phosphate ion and between ammonia and ammonium. P was removed from solution when nitrate was available, but not with ammonia. This is the case regardless of whether the starting pH was 4 or 7 (Figure 6a). *Lemna* growth, measured as dry mass, was substantially higher on nitrate. The cultures with the initial pH at 7 grew better than those where the initial pH was 4 (Figure 6c). However, the nitrate culture at pH 4 grew better than the ammonium culture at pH 7. The ammonium culture at pH 4 lost dry mass over time and became chlorotic. (Figure 6e). Only the nitrate culture at the initial pH of 7 showed substantial increase in internal Pi (Figure 6b). The pH of the medium was followed throughout the experiment (Figure 6d). The nitrate cultures with the starting pH of 7 showed only a small increase in pH to about 7.5 over the course of the experiment. In contrast, the nitrate culture with the starting pH of 4 rapidly increased in pH over the first ten days of the experiment until it converged with that of the pH 7 nitrate culture. Conversely, the ammonium pH 7 culture showed a rapid decrease in solution pH to around 3.5 over the first ten days of the experiment. The solution of the ammonium pH 4 culture did not change in pH. Control experiments without plants run in parallel (data not shown) showed that these pH changes were mediated by the plants and/or associated microbiota.

## 4 DISCUSSION

In this study, we systematically explored the effect of photoperiod, temperature, and N species on growth and P removal by *Lemna*, focusing on temperatures and photoperiods representative of seasonal variations experienced in the UK (temperate climate country). We established that significant P uptake occurs at 15°C and that while a 12‐hr photoperiod is best for Pi accumulation and growth (dry mass production), 6 hr is almost as good with both treatments removing ca 80% of the P from solution over 4 days (Figure 1). Plants maintained in 24‐hr photoperiod did not increase in biomass, but still took up and accumulated internal P showing that growth and P uptake can be uncoupled. Interestingly, even under dark conditions (0‐hr photoperiod), *Lemna* initially continued to take up P, but this stopped after 1 day, where after dry mass and internal P declined as cell constituents such as membrane lipids and proteins are degraded to support essential life processes in the absence of photosynthesis. Normally, plants photosynthesize in the light and store starch which is broken down at night for respiration and growth. In studies on starch accumulation, it was reported that inhibition of growth of *Lemna aequinoctialis* 6,000 in 24‐hr light was only observed at when light intensity exceeded 200 µmol/m^2^ s^‐1^ (Yin et al., 2015) and for *Landoltia punctata* a higher growth rate was reported in 24‐hr light at 130 µmol/m^2^ s^‐1^ compared to a 16‐hr photoperiod, although net photosynthetic rate was lower and decreased over time when plants were maintained in 24‐hr light (Liu et al., 2018). This may reflect differences in the sensitivity of the different species to light‐induced damage of the photosynthetic machinery, which is repaired during the dark period. Loss of internal Pi in dark grown plants is likely to be because it is required for respiration to maintain ATP levels in the absence of photosynthesis. Light intensity was not explored as a parameter in the current study and was constant at 155 µmol photons m^‐2^ s^‐1^, which is low relative to outside light even on a dullish day (Ritchie, 2010). However, duckweed prefer shaded environments with light saturation of photosynthesis occurring between 300 and 600 µmol photons m^‐2^ s^‐1^ (Wedge & Burris, 1982; Yin et al., 2015) so light intensity is unlikely to be limiting for growth and P uptake in temperate climates, even in winter.

To explore the interaction of photoperiod and temperature, further the photoperiod was fixed at 6 hr and temperature varied between 8 and 25°C (Figure 2). At 8°C, duckweed did not grow, consistent with previous reports (Lasfar, Monette, Monette, Millette, & Azzouz, 2007) but still removed P from solution, albeit more slowly than at higher temperatures, further reinforcing that growth and uptake can be uncoupled. Plants grown at 8°C and 6‐hr photoperiod still accumulated nearly 1% dry mass as P. *L. japonica* 0223 and *L. punctata* 0224 could grow slowly at temperatures as low as 6°C in an outdoor pilot system and had similar internal P as reported here (Zhao et al., 2015). Mass balance experiments conducted over the range of temperatures showed that even in nonaxenic systems >90% of the P removed from solution was recovered in the duckweed (Figure  3c), showing that this is an effective method of capturing solution P and that harvested duckweed could potentially be processed to recover the concentrated nutrients, for example, to use as fertilizer. The total amount of P recovered will be determined by the internal P concentration in harvested biomass. These results suggest that greater P recovery could be achieved under these conditions by higher stocking densities. The mat density has a significant impact on growth (Frédéric, Samir, Samir, Louise, & Abdelkrim, 2006) and may explain the discrepancy between our finding that photoperiod has little effect and a previous study which tested photoperiods between 2 and 20 hr and found 12 hr to be optimum with growth reduced by approximately 2/3 at 6 hr (Lasfar et al., 2007).

Intracellular P accumulation is the sum of a number of different processes including transport, metabolic utilization (incorporation into macromolecules such as nucleic acids and phospholipids), and storage of phosphate the vacuole. Direct measurements of P transport using ^32^P labeled orthophosphate showed that the transport is strongly temperature dependent (Figure 4). Transport rates approximately doubled between 5 and 15°C (2.4‐fold) and 15 and 25°C (2.8‐fold) consistent with most biological systems having a temperature coefficient (Q_10_) of between 2 and 3. The rate of transport measured at 25°C is consistent with those reported previously (Ullrich‐Eberius et al., 1984). Phosphate uptake into cells is an active transport process linked to the transmembrane potential, since phosphate is co transported into the cell with protons. Transport also requires conformational changes of transport proteins within the membrane (Pedersen et al., 2013), the fluidity of which is strongly dependent upon temperature (Murata & Los, 1997). In the experiment reported in Figure 3a, the plants had been grown at 15°C and were acclimatized to the experimental temperature for 30 min prior to the start of the experiment. A longer period of acclimatization (as would naturally occur in longer term experiments such as those in Figure 2) might increase the rate of uptake at lower temperatures as organisms adjust their membrane lipid composition as a function of temperature (Yamori, Hikosaka, Hikosaka, & Way, 2014) to maintain membrane fluidity so the rates reported here are probably lower estimates.

P uptake was correlated with P concentration in the media, with more rapid uptake at higher initial concentrations (Figure 3b). P removal coefficient (calculated from ln(e) of remaining P concentration) gave an *R^2^* of 0.8699. Intracellular P accumulation was also highly correlated with P load (*R^2^* = 0.99, Figure 3d). Measurement of ^32^P orthophosphate uptake at 22°C at a range of phosphate concentrations showed a biphasic plot consistent with low‐ and high‐affinity transport components (Figure 4b) as described previously (Ullrich‐Eberius et al., 1984). The *Spirodela* genome has three putative phosphate transporters of the PHT1 family which could encode the transporters responsible for this activity (Table S1, Figure S1) but it is not possible to say based on protein sequence conservation and modeling which are responsible for the different activities (Ceasar, Baker, Baker, Muench, Ignacimuthu, & Baldwin, 2016). Indeed, it has been reported that plant phosphate transporters may be able to switch between high‐ and low‐affinity modes (Ayadi et al., 2015). The low‐affinity transporter/mode will be important in rapidly removing solution phosphate when present above the *K*_m_ value of 0.5 mM phosphate (equivalent to 5.3 mg P L^‐1^) whereas the low‐affinity activity will be important for removing the P at concentrations between 0.2 and 5.0 mg P L^‐1^).

Plants respond to scarcity of P by activating a genetic program that increases the expression of phosphate transporters for scavenging low concentrations of P from the environment (Baker et al., 2015) and reprograms metabolism to conserve the scarce resource, for example, by substituting phospholipids with sulfolipids (Nakamura, 2013). Bacteria and yeast (*S.cerevisiae*) exhibit a phenomenon called polyphosphate overplus (Harold, 1966) and microalgae luxury phosphate uptake (Powell, Shilton, Shilton, Chisti, & Pratt, 2009) where they can acquire and store excess P following a period of starvation. While response of duckweed to P deficiency has hardly been studied, it is proposed that like plants they store inorganic phosphate in the vacuole. There are reports of upregulation of high‐affinity phosphate transporters on P deprivation (Hase et al., 2004; Ullrich‐Eberius et al., 1984). Pre‐acclimating Lemna to low levels of P to deplete internal P stores allowed subsequent more rapid P uptake even at 8°C and 6‐hr photoperiod when growth did not occur (Figure 5). These data further corroborate the lack of correlation between growth and P removal and suggest that a prototype design incorporating a P starvation stage could be beneficial in optimizing P recovery by duckweed under temperate conditions.

Finally, the effect of N species and pH on P removal was explored since the type of N species depends on the type of effluent, and for example, whether a STW is nitrifying or not. Additionally, there is inconsistency in the literature as to whether nitrate or ammonium are preferred sources of *N* (Porath & Pollock, 1981). *Lemna minor* is reported to have a broad initial pH tolerance when grown on unbuffered Jacob's media (N as nitrate) (McLay, 1976). It grew well in ammonium up to 3mM (42 mg/L) but showed oxidative stress and cell death above 4 mM (56 mg/L) (Huang et al., 2013) and downregulation of housekeeping genes and upregulation of genes involved in biosynthesis of lignin when cultured on high levels (84 and 840 mg/L) of ${\text{NH}}_{4}^{+}$ at pH5.5 (Wang, Li, Li, Zhu, Tang, & Zhao, 2016). *Lemna gibba* grew optimally on 50 mg/L ${\text{NH}}_{4}^{+}$ (Oron, Porath, Porath, & Wildschut, 1986) but ammonium and ammonia inhibited *Spirodela polyrhiza* in a pH‐dependent manner (Caicedo et al., 2000). Figure 6 shows a clear preference for nitrate over ammonium for growth, P uptake, and internal P concentration. The solution pH also exerts an important effect with nitrate at pH 7 performing best on all measures. In the nitrate pH 4 samples, pH rises steadily over the first two weeks of the experiment plateauing just about 7. Appreciable P uptake only starts when pH is above about 5.5 – 6.0 (day 6 or 7) which probably reflects availability of $H_{2}{\text{PO}}_{4}^{‐}$ and $H_{2}{\text{PO}}_{4}^{2‐}$, the preferred forms of phosphate for plants (the p*K*a for this transition is 7.2). The increase in pH will be at least in part attributable to proton linked uptake of both phosphate (Ullrich‐Eberius, Novacky, Novacky, Fischer, & Lüttge, 1981) and nitrate (Wang, Hsu, Hsu, & Tsay, 2012) into cells. In the case of ammonium, there is little or no growth and no P uptake. ${\text{NH}}_{4}^{+}$ is preferentially taken up by *Lemna* (Cedergreen & Madsen, 2002) but at high concentrations intracellular dissociation to NH_3_ effectively reduces the membrane potential (Britto, Siddiqi, Siddiqi, Glass, & Kronzucker, 2001; Caicedo et al., 2000; Ullrich, Larsson, Larsson, Larsson, Lesch, & Novacky, 1984) and inhibits uptake of other ions (Ullrich et al., 1984). Plants may expel protons (using energy from ATP hydrolysis) to maintain membrane potential in presence of ammonium uptake hence the falling solution pH in the pH 7 set which in turn reduces P availability as well as placing a drain on ATP supply. Nitrification reactions by duckweed‐associated bacteria may also contribute to the fall in pH on ammonium (Xu & Shen, 2011). At pH 4, the plants are chlorotic on ${\text{NH}}_{4}^{+}$ consistent with previous reports (Huang et al., 2013) but are green and vigorous on nitrate (Figure 6e).

## 5 CONCLUSIONS

The data presented in this study suggest that use of duckweed for wastewater remediation in temperate conditions is worthy of further exploration, particularly for small and rural communities where access to land is available and affordable. We show that active growth is not necessary for P uptake and that significant P uptake and intracellular P accumulation can occur at low temperature and short photoperiods, which can be beneficial to meet discharge consents even under typical temperate spring/autumn conditions. In that context, the use of duckweed ponds (≈ 4 days retention time) in small STWs could help reducing the footprint of the treatment system, when compared with other alternatives such as algal ponds (≈ 14 days retention time) or the net O&M costs if chemical precipitation for P control is used instead. In contrast to some studies in the literature, we find that nitrate and neutral pH are optimal for both rate of P removal from solution and intracellular P accumulation, which is highly beneficial for the overall performance of STWs as some nitrification is needed in order to help reducing organic matter concentrations to current discharge limits. We discovered a very strong correlation between internal P content and rate of subsequent P uptake, suggesting a period of P starvation (which could be imposed by maintenance in the dark or in waters with very low P) could be an element of engineering system design that requires further development. However, conditions that promote maximum internal P concentration and biomass yield may differ from those required to drive solution P concentration to levels required to meet exacting environmental standards. Therefore, the implementation of duckweed units for polishing the effluent from STWs in temperate climate countries should be considered both upstream (level of nitrification) and downstream processes (biomass harvesting and valorization for effective P recovery and reuse), as well as an engineering design that decouples liquid flow direction from biomass flow direction (from low to high P conditions).

## CONFLICT OF INTEREST

None.

## AUTHOR CONTRIBUTIONS

JBP conducted the research. AB and MACV supervised the research. All authors analyzed data and contributed to the writing of the manuscript. All authors approved the final version.

## Supporting information

Supplementary Material
