# Supplementary material for: Uncoupling growth from phosphorus uptake in Lemna: Implications for use of duckweed in wastewater remediation and P recovery in temperate climates
Source: Food Energy Secur. 2020 Aug 30;9(4):e244. doi: 10.1002/fes3.244 (PMC7757166; doi:10.1002/fes3.244)
Supplement: Supplementary file 1 — Supplementary Material [file FES3-9-e244-s001.docx]

Paterson et al., Supplementary information

Table S1 Sequence identity between the nine members of the Arabidopsis thaliana PHT1 family and the 3 putative homologues from *Spirodela polyrhiza*. The indicated Arabidopsis protein sequence was used ibn a blast query against the *Spirodela polyrhiza* genome at [www.phytozome.org](http://www.phytozome.org). The first number is the P value for significance of match the second is the % of identical amino acids between the two protein sequences.

| Query sequence |  |  |  |
| --- | --- | --- | --- |
|  | Spipo16G0039800 | Spipo1G0022900 | Spipo16G0039500 |
| AtPHT1;1 | 0.0 (72% identity) | e-180 (72% identity) | e-177 (72% identity) |
| AtPHT1;2 | 0.0 (72% identity) | e-177 (72% identity) | e-175 (71% identity) |
| AtPHT1;3 | 0.0 (77% identity) | 0.0 (76% identity) | 0.0 (78% identity) |
| AtPHT1;4 | e-175 | e-168 | e-172 |
| AtPHT1;5 | e-179 | e-171 | e-175 |
| AtPHT1;6 | e-161 | e-159 | e-156 |
| AtPHT1;7 | 0.0 (75% identity) | e-177 (73% identity) | e-180 (74% identity) |
| AtPHT1;8 | 9e-97 | 2e-96 | 8e-98 |
| AtPHT1;9 | e-104 | e-101 | e-101 |

Figure S1

CLUSTAL O (1.2.4) multiple sequence alignment of Arabidopsis PHT1;3 and Spirodela PHT1

homologues. Stars indicate amino acids conserved in all sequences, dots indicate conservative substitutions.

AtPHT1;3 MADQQLGVLKALDVAKTQLYHFTAIVIAGMGFFTDAYDLFCVSLVTKLLGRLYYFNPTSA 60

Spipo1G0022900 MARDQLKVLSALDVATTQWYHFTAIVVAGMGFFTDAYDLFCISLCTKLLGRIYYSEPGAA 60

Spipo16G0039500 MAGDQLQVLTALDVAKTQLYHFKAIVIAGMGFFTDAYDLFCISLITKLLGRIYYYEEGSD 60

Spipo16G0039800 MARDQLQVLTALDVAKTQWYHFTAIVIAGMGFFTDAYDLFCISLVTKLLGRIYYFQNDPE 60

** :** **.*****.** ***.***:**************:** ******:** :

AtPHT1;3 KPGSLPPHVAAAVNGVALCGTLAGQLFFGWLGDKLGRKKVYGITLIMMILCSVASGLSLG 120

Spipo1G0022900 KPGTLPPHVSAAVNGVAFCGTLLGQLFFGWLGDKLGRKKVYGMTLSLMVICSVASGLSFG 120

Spipo16G0039500 RPGSLPPNVAAAVNGVAFCGTLSGQLFFGWLGDKMGRKRVYGITLVIMVVCSIASGLSFG 120

Spipo16G0039800 RPGSLPPNVAAAVNGVAFCGTLSGQLFFGWLGDKMGRKKVYGMTLMLMVICSIASGLSFG 120

:**:***:*:*******:**** ***********:***:***:** :*::**:*****:*

AtPHT1;3 NSAKGVMTTLCFFRFWLGFGIGGDYPLSATIMSEYANKKTRGAFIAAVFAMQGVGILAGG 180

Spipo1G0022900 HSTKSVMATLCFFRFWLGFGIGGDYPLSATIMSEYANKKTRGGFIAAVFAMQGFGILSGG 180

Spipo16G0039500 HSPTGTMATLCFFRFWLGFGIGGDYPLSATIMSEYANKKTRGAFIAAVFSMQGFGILAGG 180

Spipo16G0039800 RSATGTMATLCFFRFWLGFGIGGDYPLSATIMSEYANKKTRGAFIAAVFAMQGFGILAGG 180

.* ...*:**********************************.******:***.***:**

AtPHT1;3 FVALAVSSIFDKKFPSPTYEQDRFLSTPPQADYIWRIIVMFGALPAALTYYWRMKMPETA 240

Spipo1G0022900 MVAIAVSAIFEHSFPAPAYADNPSASTVPEADFLWRIILMFGALPAALTYYWRMKMPETA 240

Spipo16G0039500 LVAIVVSAAFNNVYKALPFAQDPRGSTVPQADYVWRLILMFGAVPAALTYYWRMKMPETA 240

Spipo16G0039800 MVAIIVAAAFKNKYDVPAYKDDPLGSTVPQADYVWRIILMFGAIPAALTYYWRMKMPETA 240

:**: *:: *.: : : :: ** *:**::**:*:****:****************

AtPHT1;3 RYTALVAKNIKQATADMSKVLQTDLELEERVE--DDVKDPKKNYGLFSKEFLRRHGLHLL 298

Spipo1G0022900 RYTALVAKNAQQAAADMSKVLQVKIEQVQEKAERPPLENRRGEFGLFSREFARRHGFHLL 300

Spipo16G0039500 RYTALVAKNAKLAAADMSKVLNVELVEEQEKVERIT-KKDTNSFGLFSREFARRHGLHLL 299

Spipo16G0039800 RYTALVANNAKQAAADMSKVLQVEIEEEKEKVEQIV-SDPSNKFGLFTREFARRHGIHLL 299

*******:* : *:*******:..: :. .. .:***::** ****:***

AtPHT1;3 GTTSTWFLLDIAFYSQNLFQKDIFSAIGWIPKAATMNAIHEVFKIARAQTLIALCSTVPG 358

Spipo1G0022900 GTATTWFLLDIAFYSQNLFQKDIFSAIGWIPKPATMNAIEELYRIARAQTLIALCGTVPG 360

Spipo16G0039500 GTTSTWFLLDIAFYSQNLFQKDIFSAIGWIPKANTMSAIEEVFKIARAQTLIALCGTVPG 359

Spipo16G0039800 GTTTTWFLLDIAFYSQNLFQKDIFSAIGWIPKANTMNAIEEVFRIARAQTLIALCGTVPG 359

**::**************************** **.**.*:::***********.****

AtPHT1;3 YWFTVAFIDIIGRFAIQLMGFFMMTVFMFAIAFPYNHWILPDNRIGFVVMYSLTFFFANF 418

Spipo1G0022900 YWFTVALIDRIGRFRIQIMGFFFMTVFMLALAIPYRHWAEPSHHVGFVVMYGLTFFFANF 420

Spipo16G0039500 YWFTVGLIDIMGRFAIQLMGFFFMTVFMLALAIPYNHWTH--NQIGFVVMYGLTFFFANF 417

Spipo16G0039800 YWFTVGLIDVVGRFAIQLMGFFFMTVFMLALAIPYNHWTQPDNRIGFVVMYGLTFFFANF 419

*****.:** :*** **:****:*****:*:*:**.** :::******.********

AtPHT1;3 GPNATTFIVPAEIFPARLRSTCHGISAATGKAGAIVGAFGFLYAAQPQDKTKTDAGYPPG 478

Spipo1G0022900 GPNSTTFIVPAEIFPARLRSTCHGISAASGKLGAIVGSFGFLYAAQSQDKTKTDPGYPAG 480

Spipo16G0039500 GPNSTTFIVPAEIFPARLRSTCHGISAATGKAGAIIGSFGFLYAAQNQDKTKTDRGYPPG 477

Spipo16G0039800 GPNSTTFIVPAEIFPARLRSTCHGISAAAGKAGAIIGSFGFLYAAQNKDKTKTDPGYPAG 479

***:************************:** ***:*:******** :****** *** *

AtPHT1;3 IGVKNSLIMLGVINFVGMLFTFLVPEPKGKSLEELSGEAEVDK----------------- 521

Spipo1G0022900 IGVRNSLFVLAACNLLGLLFSFLVPESKGKSLEEMSGEDQDDGEQAPASDPRLNNRTIPI 540

Spipo16G0039500 IGVKNSLFVLAVCNVLGLLFTFLVPESKGRSLEDLSGENEQSEQTEQERPPIT----FHV 533

Spipo16G0039800 IGVRNSLFVLAACNMLGFLFTFLVPESNGKSLEEMSGENEQEESETANAATAYANRTIPV 539

***:***::*.. *.:*:**:***** :*:***::*** : .
